# Novel Resolution of Multilayered Pigment Epithelial Detachment Lamellae Following Brolucizumab Treatment—A Case Report

**DOI:** 10.1155/crop/9953015

**Published:** 2025-03-06

**Authors:** Unnikrishnan Nair, Indu J. Nair, Jay U. Sheth, Manoj Soman

**Affiliations:** ^1^Department of Vitreoretinal Services, Chaithanya Eye Hospital and Research Institute, Trivandrum, India; ^2^Department of Research, Chaithanya Innovation in Technology and Eye Care (Research), Trivandrum, India; ^3^Department of Retina Services, Shantilal Shanghvi Eye Institute, Mumbai, India

**Keywords:** brolucizumab, lamellae, multilayered pigment epithelial detachment, polypoidal choroidal vasculopathy

## Abstract

**Purpose:** The aim of this study is to report a unique case where brolucizumab administration resolved multilayered pigment epithelial detachment (MLPED) lamellae.

**Observations:** An 80-year-old gentleman with polypoidal choroidal vasculopathy developed MLPED from long-term ranibizumab treatment. Switching to brolucizumab led to visual acuity improvement after three doses and complete resolution of fluid, reduced choroidal thickness, and MLPED collapse. Notably, the patient experienced a recurrence of MLPED, which again resolved after the fourth dose of brolucizumab.

**Conclusions and Importance:** This case underscores the effectiveness of brolucizumab in resolving MLPED lamellae, a previously unreported phenomenon. Furthermore, it highlights the potential for visual acuity improvement despite MLPED resolution. Brolucizumab's mechanism of action, including its potent antivascular endothelial growth factor properties and enhanced tissue penetration, may contribute to the collapse of MLPED by modulating subretinal pigment epithelial fluid dynamics. Further research into molecular pathways, cellular interactions, and safety profiles is warranted to optimize the therapeutic role of brolucizumab.

## 1. Introduction

Over the last two decades, anti-VEGF (vascular endothelial growth factor) agents have significantly transformed the management of chorioretinal diseases. Brolucizumab (Pagenax Novartis India Ltd Mumbai, India), a humanized monoclonal single-chain variable fragment (scFv), is among the approved anti-VEGF agents [1, 2]. Its mechanism involves binding and inhibiting VEGF-A, thereby impeding VEGF receptor activation and, decreasing chorioretinal neovascularization [1]. FDA approvals for brolucizumab were obtained for neovascular age-related macular degeneration (nAMD) in 2019 and for diabetic macular edema (DME) in 2022 [1, 2].

Various types of pigment epithelial detachments (PEDs) are associated with AMD and polypoidal choroidal vasculopathy (PCV) [3]. Multilayered pigment epithelial detachment (MLPED) is a PED with organized layers of hyperreflective bands/laminations between the retinal pigment epithelium (RPE) and Bruch's membrane, arranged parallel to the RPE in a fusiform spindle shape and are accompanied by a hyporeflective space known as the prechoroidal cleft [4]. Multilayering, observed as a consequence of chronic anti-VEGF therapy, is thought to encompass both fibrovascular and fibrocellular components [4, 5].

Herein, we report a case of a patient undergoing long-term anti-VEGF treatment for PCV who, following intravitreal injection of brolucizumab, had increased visual acuity and a collapse of MLPED lamellae.

## 2. Case Presentation

**Figure 1:**
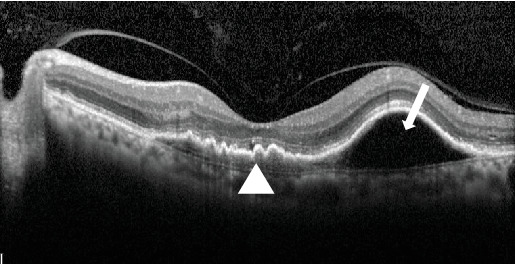
Optical coherence tomography of the left eye at baseline illustrating the flat-irregular pigment-epithelial detachment (PED) in the subfoveal region (white arrowhead) with a large PED temporally (white arrow).

An 80-year-old gentleman presented with decreased vision in both eyes for 5 years in 2014. No significant systemic history was present. His best-corrected visual acuity (BCVA) was 20/400 in the right eye (OD) and 20/120 in the left eye (OS). He had bilateral pseudophakia with normal intraocular pressure (IOP). Fundus examination showed a scarred macular neovascularization (MNV) in the OD and the presence of an active MNV in the OS. Optical coherence tomography (OCT) demonstrated a flat-irregular PED in the subfoveal region with a large PED temporally (Figure 1). A provisional diagnosis of PCV was made, and the patient received 28 intravitreal ranibizumab injections over 6 years on a pro re nata (PRN) basis. A gradual multilayering was observed over the treatment course. Subsequently, the patient was lost to follow-up (between 2020 and 2022) and presented with a recurrence.

**Figure 2:**
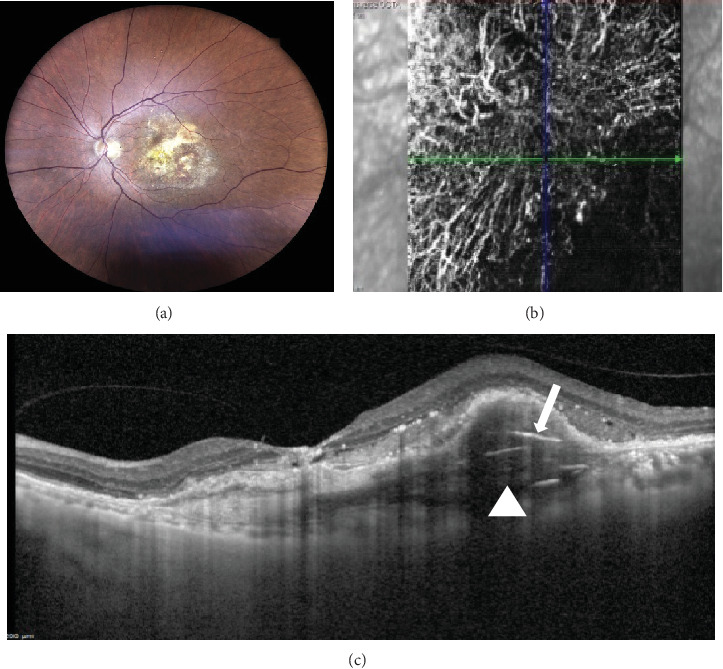
(a) Fundus photography of the left eye demonstrating the exudative maculopathy. The optical coherence tomography (OCT) at this stage illustrated the multilayered pigment epithelial detachment (MLPED) (b; white arrowhead) with hyperreflective lamellae (b; white arrow). (c) The macular neovascularization (MNV) network was confirmed on the OCT angiography.

**Figure 3:**
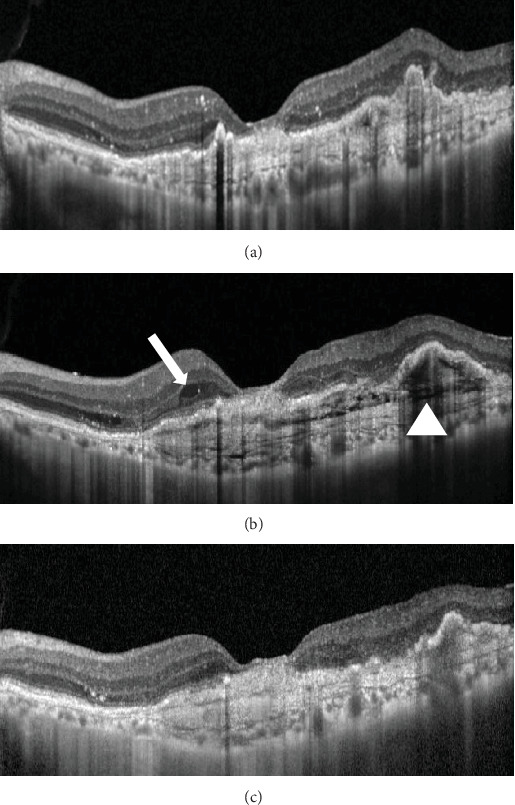
After administering three consecutive monthly doses of brolucizumab, there was a notable complete collapse of the multilayered pigment epithelial detachment (MLPED) lamellae (a). However, at 4 months, a recurrence of MLPED was observed (b; white arrowhead), accompanied by intraretinal fluid (b; white arrow) for which the patient received the fourth dose of brolucizumab. Four months thereafter, a subsequent collapse of the MLPED was observed (c).

At this stage, his BCVA was 20/80 in the OS, with exudative maculopathy on fundus examination (Figure 2a). The OCT demonstrated the presence of an MLPED along with subretinal fluid (SRF) and intraretinal fluid (IRF), hyperreflective lamellae, and thickened choroid in the OS (Figure 2c). The network was confirmed on an optical coherence tomography angiography (OCTA) (Figure 2b). The patient was switched to intravitreal brolucizumab and received three loading doses of the agent between February and April 2023. Subsequently, his BCVA improved to 20/40. The OCT demonstrated complete resolution of the IRF and the SRF with a reduction in the choroidal thickness from 430 to 297 *μ*m (Figure 3a). Additionally, a complete collapse of the MLPED lamellae was noted, too, at this stage (Figure 3a). After 4 months of MLPED collapse, the lamellae recurred with the appearance of IRF (Figure 3b) and the BCVA dropping to 20/80, for which the patient received the fourth dose of brolucizumab. Subsequently, 4 months later, the MLPED lamellae collapsed without any disease activity and a BCVA of 20/30 (Figure 3c). No ocular or systemic adverse events were noted at any visit.

## 3. Discussion

This case report introduces two pivotal observations: First, the unique resolution of the MLPED lamellae due to brolucizumab, and second, the potential improvement in visual acuity even after MLPED resolution. Additionally, the patient experienced a recurrence of MLPED, which resolved after the fourth brolucizumab dose.

AMD and PCV are associated with diverse types of PEDs, and the occurrence of multilayering often arises as a consequence of chronic anti-VEGF therapy [4, 5]. MLPEDs are characterized by a hyporeflective space termed a “prechoroidal cleft,” which separates the fusiform complex from the underlying choroid [4, 5]. This separation could stem from contraction, fluid exudation, or a combination of both factors. Studies by Rahimy et al. [4] have shown that eyes with MLPED tend to maintain good visual acuity and exhibit a lower probability of developing RPE tears [5–7]. The proposed mechanism for preserving good visual acuity involves the suppression of neovascular and cicatricial processes within the sub-RPE space due to chronic anti-VEGF therapy [5]. This preservation allows for the viability of the photoreceptor population by safeguarding the integrity of the RPE [5–7]. Multilayered PEDs have been observed in patients receiving as few as three injections to as high as 80 intravitreal injections [4].

In contrast to prior literature, Soman et al. [5] reported the occurrence of de novo MLPED in both AMD and PCV. Among the 17 eyes with MLPED in their study, 7 eyes exhibited de novo MLPED [5]. The authors concluded that MLPED represents a unique form of cicatrizing fibrovascular PED, which can evolve de novo [5]. This occurrence is identified as a consequence of prolonged disease duration characterized by intermittent or low-grade activity [5]. In comparison to MLPED eyes under chronic anti-VEGF therapy, eyes with de novo MLPED demonstrate greater visual stability despite receiving fewer anti-VEGF treatments [5].

Notably, the observation that visual acuity improves even after the resolution of MLPED in our case is indeed intriguing and adds a nuanced perspective to the disease process of AMD and PCV. While the collapse of the temporal MLPED is an important finding, we acknowledge that the resolution of fluid in the foveal region likely played a more direct role in driving the observed visual acuity improvement. This was evident both after the initial three doses and during recurrence treated with brolucizumab. This suggests that MLPED resolution may not necessarily be detrimental to the overall disease trajectory. In fact, it could indicate a positive response to treatment, potentially reflective of improved retinal and inner choroidal architecture and function. This phenomenon underscores the complexity of the pathophysiology involved and highlights the need for further research to elucidate the underlying mechanisms driving visual improvement beyond MLPED resolution. Additionally, these findings prompt a reevaluation of the role of MLPED in the context of disease progression and treatment outcomes in AMD and PCV.

Our case highlights a unique therapeutic observation. Over a prolonged course of 28 ranibizumab injections administered on a PRN basis spanning 6 years, the MLPED remained unaltered despite repeated anti-VEGF therapy. Following a period of treatment interruption, the patient's recurrence was managed with brolucizumab. Remarkably, after three loading doses of brolucizumab, there was a complete collapse of the MLPED, accompanied by significant improvements in BCVA and anatomical parameters on OCT. Furthermore, when the MLPED lamellae reappeared along with IRF and a decline in BCVA, administration of a subsequent dose of brolucizumab again resulted in collapse of the lamellae and restoration of visual acuity. This consistent response with brolucizumab—contrasted with the lack of response during the extensive ranibizumab treatment—suggests that brolucizumab may exert a distinct pharmacologic effect, potentially attributable to its smaller molecular size, higher molar concentration, and superior tissue penetration. While we recognize that the treatment gap could theoretically contribute to the observed effects, the reproducible anatomical response with brolucizumab administration indicates a specific therapeutic role in inducing MLPED collapse. Future studies are warranted to further elucidate the mechanisms underlying this response and to compare the efficacy of brolucizumab directly with other anti-VEGF agents in similar clinical scenarios.

Additionally, we recognize that a prechoroidal cleft is often observed in the imaging of MLPED and is an essential component of the diagnostic process, which we have now highlighted in the revised manuscript. Furthermore, while only a portion of the MLPED outside of the fovea appears to have collapsed or resolved, a much larger area of MLPED extends throughout the macula and beneath the fovea. This observation likely reflects chronic low-grade activity during the time the patient had been lost to follow-up. The mechanism of MLPED formation is thought to involve alternating periods of CNVM suppression and activity, potentially allowing a fibrocellular cap to form over the CNVM. This cap may serve as a protective mechanism.

Interestingly, brolucizumab appears to offer more ability to modify acute MLPED, but its effect on more chronic MLPED may be less pronounced. This differential response may be due to the plasticity of the subretinal morphological changes. In other words, while brolucizumab may effectively resolve more acute components of MLPED, the more established areas may remain unchanged, possibly due to their chronic nature. This observation adds to the growing understanding of how brolucizumab may influence the subretinal architecture in a dynamic and disease-specific manner.

Anti-VEGF therapy has emerged as the cornerstone in the management of AMD and PCV. Several randomized controlled trials have largely demonstrated the efficacy of various anti-VEGF molecules such as bevacizumab, ranibizumab, and aflibercept in MNV [8–10]. scFvs, the smallest functional unit of antibodies, offer advantages such as enhanced molar dose and improved tissue penetration, designed to prolong therapeutic effects as compared with larger molecules [11]. Additionally, this enhanced penetration potentially enables brolucizumab to reach and affect the sub-RPE space more efficiently, addressing the fluid dynamics associated with PEDs, including MLPED [3]. Likewise, brolucizumab's compact scFv structure facilitates enhanced binding efficiency to VEGF-A, which might result in a more potent suppression of the vascular leakage and subsequent accumulation of sub-RPE fluid, thus aiding in MLPED resolution. Comparative studies between brolucizumab and aflibercept revealed brolucizumab's efficacy in reducing IRF, SRF, and sub-RPE fluid [12]. The HAWK and HARRIER studies concluded noninferiority of brolucizumab to aflibercept in visual function at Week 48, with anatomical outcomes favoring brolucizumab [12]. Ogura et al. [13], in a study on PCV in Japanese eyes, observed greater fluid resolution with brolucizumab compared to aflibercept, maintaining through Week 96. Another study on the Asian population reported a 93.3% complete resolution of polypoidal lesions at 1-year post-brolucizumab injection, with an average of 6.4 ± 0.13 injections in 1 year [14].

Resolution of serous and drusenoid PEDs following brolucizumab injection has been reported [3, 15]. Chakraborty and Umed [3], in a case series involving extra-large PED (maximum height > 350 *μ*m) due to untreated MNV, reported significant PED height improvement by Week 4, with complete resolution by Week 8 in two of the three patients. Saitta et al. [15] reported the collapse of drusenoid PED after two monthly brolucizumab injections in a patient unresponsive to intravitreal bevacizumab and aflibercept. To date, no case of the collapse or resolution of MLPED has been reported in the literature. Brolucizumab's ability to disrupt fibrovascular and fibrocellular components within the sub-RPE space may contribute to the observed collapse of MLPED in our case. Further research is required to validate these findings, as the current report is constrained by its nature as a single case study.

Furthermore, it is important to consider that while rapid resolution of PEDs is generally seen as a favorable anatomical response, this swift collapse may also predispose the RPE to mechanical stress, potentially leading to an RPE tear. The abrupt retraction of fibrovascular and fibrocellular components beneath the RPE could generate forces that exceed the tissue's tensile strength, especially in eyes with large or chronic PEDs. Although our patient did not experience an RPE tear, this potential risk underscores the need for cautious interpretation of rapid PED collapse following brolucizumab treatment. Future studies with larger cohorts are warranted to better delineate the incidence and underlying risk factors associated with RPE tears in this clinical context.

Intraocular inflammations (IOIs) pose a concern in brolucizumab therapy. Ogura et al. [13] demonstrated an overall well-tolerated safety profile, albeit with a higher rate of IOI compared with aflibercept. While rates of ocular adverse effects remain similar between brolucizumab and aflibercept, brolucizumab exhibits an increased risk of inflammatory events occurring in 4%–10% of injections [16, 17] While sterile IOI has been reported after intravitreal injection of other anti-VEGFs at an estimated rate between 0.3% and 2.9% per injection, IOI was noted in 4.4% of brolucizumab-treated eyes in the HAWK and HARRIER studies [16, 18]. In our case, no IOI was observed after any of the four brolucizumab injections.

In conclusion, this case report underscores the promising efficacy of brolucizumab in resolving MLPED lamellae and enhancing visual acuity postresolution, even following recurrence. Improvements in visual acuity postresolution challenge conventional understanding, suggesting a positive treatment response. Further research could delve deeper into the specific molecular pathways and cellular interactions at the sub-RPE and choroidal levels underlying these actions, refining our understanding of brolucizumab's role in treating such complex chorioretinal pathologies.

## Data Availability

The data that support the findings of this study are available from the corresponding author upon reasonable request.

## References

[1] Chakraborty D., Maiti A., Sheth J. U. (2022). Brolucizumab in neovascular age-related macular degeneration – Indian real-world experience: the BRAILLE study – fifty-two-week outcomes. *Clinical Ophthalmology*.

[2] Chakraborty D., Maiti A., Sheth J. U. (2021). Brolucizumab in neovascular age-related macular degeneration-Indian real-world experience: the BRAILLE study. *Clinical Ophthalmology*.

[3] Chakraborty S., Umed S. J. (2023). Response of extra-large pigment epithelial detachment to intravitreal brolucizumab injection. *American Journal of Ophthalmology Case Reports*.

[4] Rahimy E., Freund K. B., Larsen M. (2014). Multilayered pigment epithelial detachment in neovascular age-related macular degeneration. *Retina*.

[5] Soman M., Sheth J. U., Indurkar A., Meleth P., Nair U. (2021). De-novo multilayering in fibrovascular pigment epithelial detachment. *Scientific Reports*.

[6] Au A., Hou K., Dávila J. P. (2019). Volumetric analysis of vascularized serous pigment epithelial detachment progression in neovascular age-related macular degeneration using optical coherence tomography angiography. *Investigative Ophthalmology & Visual Science*.

[7] Christenbury J. G., Phasukkijwatana N., Gilani F., Freund K. B., Sadda S., Sarraf D. (2018). Progression of macular atrophy in eyes with type 1 neovascularization and age-related macular degeneration receiving long-term intravitreal anti-vascular endothelial growth factor therapy: an optical coherence tomographic angiography analysis. *Retina*.

[8] Rosenfeld P. J., Brown D. M., Heier J. S. (2006). Ranibizumab for neovascular age-related macular degeneration. *New England Journal of Medicine*.

[9] CATT Research Group (2011). Ranibizumab and bevacizumab for neovascular age-related macular degeneration. *New England Journal of Medicine*.

[10] Heier J. S., Brown D. M., Chong V. (2012). Intravitreal aflibercept (VEGF trap-eye) in wet age-related macular degeneration. *Ophthalmology*.

[11] Dugel P. U., Jaffe G. J., Sallstig P. (2017). Brolucizumab versus aflibercept in participants with neovascular age-related macular degeneration: a randomized trial. *Ophthalmology*.

[12] Dugel P. U., Koh A., Ogura Y. (2020). HAWK and HARRIER: phase 3, multicenter, randomized, double-masked trials of brolucizumab for neovascular age-related macular degeneration. *Ophthalmology*.

[13] Ogura Y., Jaffe G. J., Cheung C. M. G. (2022). Efficacy and safety of brolucizumab versus aflibercept in eyes with polypoidal choroidal vasculopathy in Japanese participants of HAWK. *British Journal of Ophthalmology*.

[14] Ito A., Maruyama-Inoue M., Kitajima Y., Ikeda S., Inoue T., Kadonosono K. (2022). One-year outcomes of intravitreal brolucizumab injections in patients with polypoidal choroidal vasculopathy. *Scientific Reports*.

[15] Saitta A., D'Eliseo L. A., D'Eliseo D. (2023). Efficacy and safety of brolucizumab for serous drusenoid pigment epithelium detachment non-responder to bevacizumab and aflibercept. *European Journal of Ophthalmology*.

[16] Monés J., Srivastava S. K., Jaffe G. J. (2021). Risk of inflammation, retinal Vasculitis, and retinal occlusion–related events with brolucizumab: post hoc review of HAWK and HARRIER. *Ophthalmology*.

[17] Baumal C. R., Spaide R. F., Vajzovic L. (2020). Retinal vasculitis and intraocular inflammation after intravitreal injection of brolucizumab. *Ophthalmology*.

[18] Chakraborty D., Stewart M. W., Sheth J. U. (2021). Real-world safety outcomes of intravitreal ranibizumab biosimilar (Razumab) therapy for chorioretinal diseases. *Ophthalmology and Therapy*.

